# The impact of implantation site on procedure success in patients with unresolved facial palsy treated with upper-eyelid gold weight loading

**DOI:** 10.1038/s41598-022-16169-4

**Published:** 2022-07-13

**Authors:** Izabela Nowak-Gospodarowicz, Robert Koktysz, Marek Rękas

**Affiliations:** 1 Department of Ophthalmology, Military Institute of Medicine, 128 Szaserow St., 04-141 Warsaw, Poland; 2 Department of Pathology, Military Institute of Medicine, 128 Szaserow St., 04-141 Warsaw, Poland

**Keywords:** Medical research, Pathogenesis, Risk factors, Signs and symptoms

## Abstract

Loading of the upper eyelid is a well-established procedure for the correction of incomplete eyelid closure due to unresolved facial palsy. Some incurable complications are attributed to type IV hypersensivity reaction, but there is no confirmation of this hypothesis. The aim of the study was to show the impact of gold weights on eyelid tissues depending on the implantation site. Out of 94 total patients (aged 53 ± 17 years) treated from July 2009–2021, since 2014 thirty consecutive patients were randomised into one of 3 groups: the GLE group (gold weight fixed 2 mm above the eyelash line), the GUE group (gold weight fixed at the border of the tarsus and the levator aponeurosis), and the PUE group (platinum chain fixed in the same way as in the GUE group). In the cases of complications, the explanted weights were evaluated histopathologically. The outcomes were compared between groups. Incomplete eyelid closure was corrected in all patients. Serious complications were noted in 100% of patients in the GLE group and 20% in the GUE group (p < 0.0001). A slight lymphocytic reaction was observed in the GUE group. A moderate to significant lymphocytic reaction was observed in the GLE group (p < 0.001). Adverse reactions of the upper eyelid microenvironment resulting from gold weights seem to be dependent on mechanical damage to the eyelid structures, rather than on implants themselves. The site of placement of the weight in the upper eyelid may be critical for procedure success.

## Introduction

Facial palsy (FP) may have many etiologies, though luckily most patients recover without any serious sequelae. However, some patients have irreversible FP which is a challenge for specialists in many fields of medicine. These are usually patients with a tumor of the cerebellopontine angle, patients who have had surgeries to remove a primary tumor, patients who have had resection of salivary gland tumors or patients with a history of trauma^1^. Although these patients usually have a good prognosis as to their main disease, complications related to facial nerve dysfunction seem to be of the utmost importance. The inability to close the eye leads to its drying out and the development of severe corneal complications resulting in reduced visual acuity, decreased quality of life and even perforation and loss of the eye^2,3^. The idea of adding weight to the non-closing upper eyelid with a gold weight was first presented by K. Illig in 1958^4^. Smellie GD popularized this method in English-language writings and gold was introduced as the metal of choice for loading of the upper eyelid^5^.

The following years brought a wealth of alternative surgical techniques to resolve incomplete eyelid closure^6–11^. However, due to the difficulty of these surgical techniques, the cost of matarials and the high percentage of complications, loading of the upper eyelid with a gold weight was resumed as it is a relatively simple, effective and safe technique. With further modifications of implants and operation techniques^12–16^, gold weights have become a widely used and effective method for the correction of lagophthalmos in established FP^17–22^*.* Complications related to implantation are relatively rare and include: weight extrusion, migration, bulging, induced astigmatism responsible for deterioration of visual acuity, under- or overcorrection and unsatisfactory cosmesis^23–26^*.* Only a few cases of presumed allergic or other non-infectious inflammatory reaction to gold weights have been reported in the literature as a serious incurable complication, which necessitates the removal of the implant^27–33^.

Some late complications are attributed to type IV hypersensivity reaction^27,31–33^*.* However*,* because of the low number of patients in the study groups, uncertain accuracy of screening tests, scarce histopathological examination and limited immunohistochemical typing of the inflammatory cells, there is still no direct confirmation of this hypothesis. Gold was considered biologically inert through the late 1980s, when 0.5% sodium thiosulfate was introduced as a good screening test in the diagnosis of a contact allergy to gold^34^. After a number of initial reports on the high frequency of allergy to gold diagnosed on the basis of patch testing with gold sodium thiosulfate, it was proven that the patch test alone is not sufficient to confirm a contact allergy^35,36^. According to the study of Anhilide et al. patch testing with gold sodium thiosulfate alone has a positive predictive value of only 40%^37^.

The mechanisms responsible for the development of symptomatic allergic contact dermatitis are still under investigation^38^. The innate immune system can be activated by metal ions or other chemicals at the tissue/material interface, which causes disfunction of the immune system and activation of the double immune response (allergen-specific effector and memory Tcells mediated). However, there are many host-and biomaterial-specific factors which can modify the autologous innate stimulus, which, in turn, can influence individual susceptibility to an allergen^39^. Except for the innate immune response, a variety of different types of proteins in the coagulation, complement, fibrinolytic and kinin-generating systems and platelets are activated in the processes of simultaneous injury and wound healing following implantation of biomaterials^40^. Such explanations do not consider placement site as a factor. The aim of this study was to investigate the late impact of gold implants on the tissues of the upper eyelid depending on the implantation site. After conducting a case study in order to find possible causes of complications after loading of the upper eyelid with gold weights, we conclude that weight placement site in the upper eyelid, rather than a gold allergy itself, may be critical for procedure success^41^. In the years since this initial reporting, we conducted a prospective study in order to confirm or reject this theory. This paper reports on new findings derived from follow-up research.

## Material and methods

### Study design

This research was part of a prospective, interventional study that assessed clinical outcomes in patients after weight implantation in the upper eyelid as treatment for ocular complications of unresolved FP between July 2009 and July 2021. The study was approved by the designated ethics committee (Bioethical Commission at the Military Institute of Medicine in Warsaw; ethical approval number 57/WIM/2011, received on 17 August 2011) and adhered to the tenets of the Declaration of Helsinki. All patients provided written informed consent to participate in this study. Informed consent was obtained to publish information/images in an online open access publication.

### The study groups

Out of 94 total patients (mean age 53 ± 17 years) treated at our clinic with upper eyelid loading between July 2009 and July 2021, since the year 2014 thirty consecutive patients were randomised into one of 3 groups: Patients from the first group (GLE) received a gold weight fixed to the tarsus 2 mm above the upper eyelid margin. In the second group (GUE), the gold weight was fixed at the border of the upper edge of the tarsus of the upper eyelid and the levator aponeurosis. Patients in the third group (PUE) received a platinum chain fixed in the same way as in the second group (GUE). The inclusion criteria were: (1) Unresolved FP and unchanged lagophthalmos for at least 3 months despite intensive rehabilitation, (2) ocular symptoms reported by the patient due to exposure keratopathy not responding to conservative treatment (topical treatment with moisturizers and eye patching or moist chamber), (3) at least good function (> 4 mm) of the levator muscle of the upper eyelid, and (4) condition of both the skin of the eyelid and orbicularis oculi muscle allowed for surgery. Estimation of the implant weight, the surgical technique and the evaluation process have been reported in detail elsewhere^2,3^.

**Figure 1:**
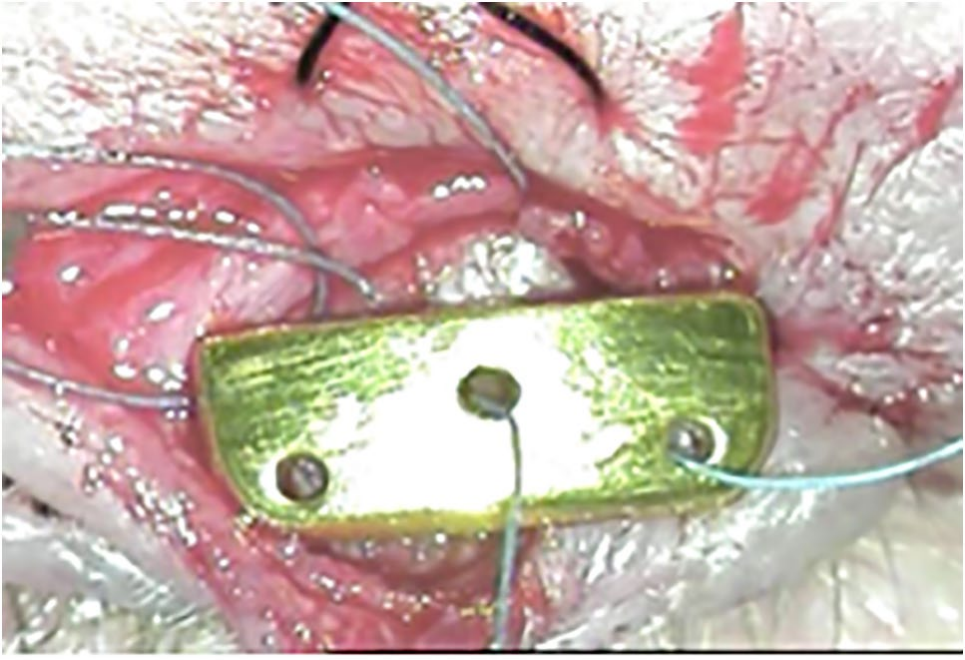
Typical implantation site for a gold weight.

Detailed medical histories of previous and concomittant diseases were collected with special attention to their etiology, duration, method and efficacy of treatment. All patients were asked about symptoms, personal and family history of any allergy, wearing gold jewellery, dental restoration involving gold and the use of cosmetics containing gold. Thereafter, a full ophthalmic examination was performed. After obtaining patient consent, all findings were documented by means of standard macroscopic and microscopic pictures. All surgeries were performed by a single surgeon (IN-G) under a microscope and local anaesthesia with infiltration of a lidocaine, adrenaline and hyaluronidase mixture. Patients were washed and draped. The skin was incised in the eye crease with a surgical scalpel blade No.15. The orbicularis oculi muscle was identified and carefully separated from the skin. First, the muscle was incised transversely, parallel to the lid margin, on the level of the levator aponeurosis muscle insertion to the tarsus. Then, dissecting sccissors were used to dissect it deeper and downwards to the tarsus. A sterile weight was sutured to the anterior surface of the tarsus with absorbable polyglactin sutures (Vicryl 6–0) (Fig. 1). The GLE group received a gold weight fixed about 2 mm above the eyelash line. In the GUE group, the gold weight was fixed at the border of the upper tarsus and the levator aponeurosis. The PUE group received a platinum chain fixed in the same way as in the GUE group. Absorbable sutures were used to avoid inducing long term foreign body giant cell reactions, which were observed near the non-absorbable suture in our previous study^41^. Coagulation was avoided in order not to harm the affected tissues. In concomittant retraction of the upper eyelid the anterior part of the levator aponeurosis muscle was dissected from the tarsus. In cases of extrusion or other complications which required us to remove the implant from the upper eyelid, a typical implant-size-dependent capsule that formed between the orbicularis muscle and the anterior surface of the tarsus was identified. Special care was taken to remove the whole capsule with the implant inside. The muscle was sutured with the same absorbable sutures. The skin was pieced together with silk, non-absorbable sutures (Mersilk 6–0). The wound was covered by a sterile dressing with antibiotic and steroid ointment and left for 24 h. As part of standard postoperative care, all patients received a 2-week course of topical antibiotic and steroid ointment (oxytetracycline with hydrocortisone) 4qid (4 times a day) and topical lubricants, as needed. The skin sutures were removed on postoperative day 8**.** In the cases of extrusion the same sterilized implant was placed under the orbicularis muscle at the border of the upper edge of the tarsus of the upper eyelid and the levator aponeurosis.

### Histopathology

The surgically explanted weights with surrounding tissues were sent to the Department of Pathology, where tissues were removed and divided into four parts according to their localization (frontal upper and lower and posterior upper and lower). Each specimen was separately processed on a paraffin block, cut and stained with the hematoxylin—eosin method. Specimens were also stained with the routine Masson trichrome method to differentiate soft tissues. Immunohistochemical stains were performed using CD3, CD20, CD4, CD8 and Mac387 monoclonal antibodies (DAKO; immunoperoxidase method—DAKO autostainer). Proportion scores of CD3/CD20 and CD4/CD8 were calculated to qualify the type of lymphocytic reaction. All specimens were evaluated by the same experienced pathologist (RK).

The pathologist was not informed about the weight placement site in the upper eyelid.

The histopathology results were combined with the clinical findings.

### The statistical analysis

Statistical analysis was performed using SPSS software (IBM Corp. Released 2012. IBM SPSS Statistics for Windows, Version 21.0. Armonk, NY: IBM Corp, USA). For measurable features, the normality of the distribution of analyzed parameters was evaluated using the Shapiro–Wilk test. The Wilcoxon pair order test was used to compare the two dependent groups. For more than two independent groups, the Kruskal–Wallis test was used. A significance level of *p* < 0.05 was considered significant.

## Results

**Figure 2:**
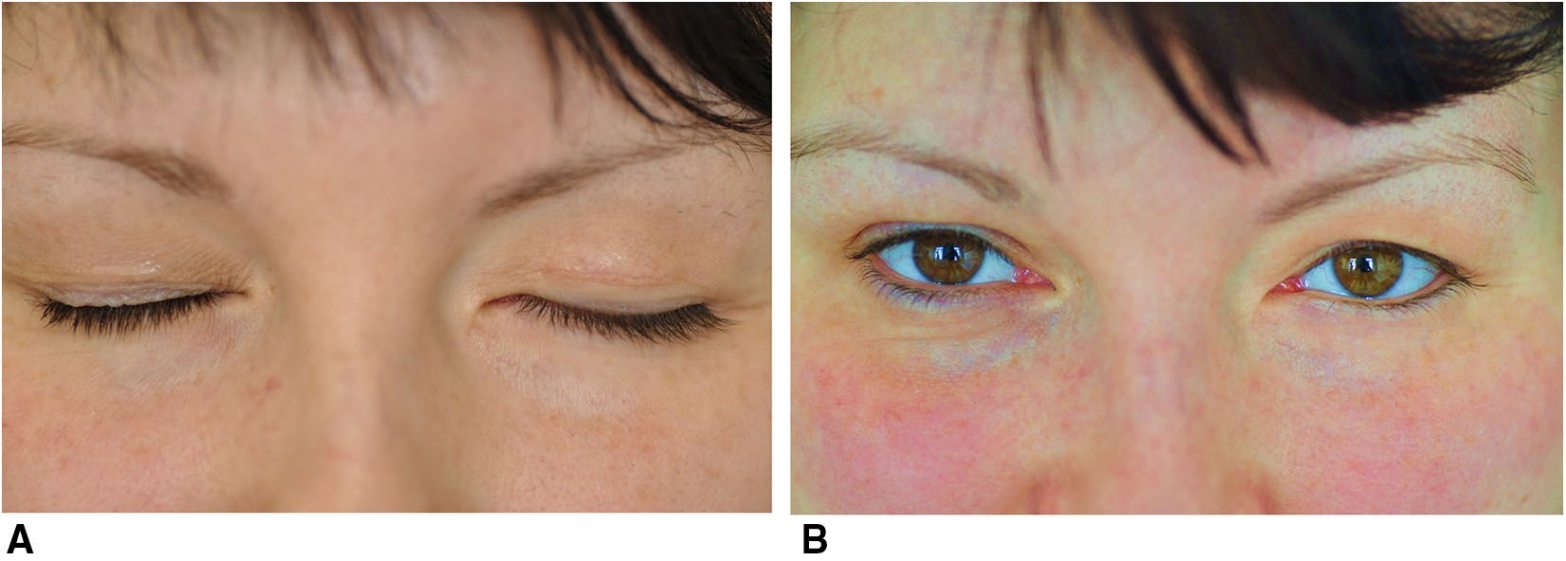
Optimal result in 37 year old patient after upper eyelid gold weight loading in left eye at a 6 month follow-up. (**a**) Patient at a 6-month follow-up after loading of the left upper eyelid with a gold weight (1.4 g) with closed eyes. (**b**) Patient at a 6 month follow-up after loading of the left upper eyelid with the gold weight (1.4 g) in primary gaze.

There was no known personal history of any metal allergy in the study group. Incomplete eyelid closure was corrected in every patient from the study group as per the primary treatment approach (Fig. 2a,b). Lagophthalmos was on average 7 ± 3 mm in the GLE group, 7 ± 3 mm the GUE group and 8 ± 4 mm in the PUE group prior to surgery (p > 0.1). The average weight of the implant was 1.5 ± 0.3 g in the GLE group, 1.6 ± 0.2 g in the GUE group and 1.6 ± 0.4 mm in the PUE group (p > 0.1). Twelve patients (9 women and 3 men) aged 21–79 years (mean age 49 ± 17 years) required gold weight removal due to postsurgical complications (100% in the GLE group, 20% in the GUE group, 0% in the PUE group, p < 0.0001). Gold weights had to be removed because of: extrusion in 6 patients (60% of the GLE group), presumed allergy to gold manifested by reddened swollen eyelid in 3 patients (30% of the GLE group), unsatisfactory cosmesis in 2 cases (10% of the groups GLE and GUE) and excessive ptosis in 1 patient (10% of the GUE group). In the PUE group we observed only slight reddened eyelid in one case at a 1-month follow-up, but the patient refused to have the implant removed from the eyelid tissues.

All complications that required gold weight removal were observed at 5–84 postoperative months (in the GLE and GUE groups). The mean explanted implant weighed 1.5 ± 0.3 g.

The basic cinical data are presented in Table 1. All explanted weights were surrounded by fibrotic capsules with lymphocytic infiltrates CD3, CD4, CD8, and CD20 positive.

**Table 1:** Clinical data.

Patient	Age	Gender	Eye	Etiology of facial palsy	Weight [g]	Cause of implant removal	Clinical signs	Time from surgery to implant removal [months]	Previous eyelid surgery	Implantation site
1	24	F	L	ANS	Gold [1.4]	extrusion	reddened mildly oedematous lid with visible vessels and thin orbicularis muscle layer	5	Yes	GLE group
2	35	M	R	ANS	Gold [1.4]	extrusion	reddened and mildly oedematous lid with visible blood vessels	82	Yes	GLE group
3	42	M	L	ANS	Gold [1.2]	extrusion	slightly reddened with visible blood vessels	31	Yes	GLE group
4	37	F	L	Trauma	Gold [1.4]	cosmesis	asymptomatic pale eyelid	12	No	GUE group
5	60	F	R	ANS	Gold [1.0]	extrusion	slightly reddened with vessels visible on the surface of the skin	29	No	GLE group
6	75	F	L	HZO	Gold [1.8]	ptosis	pale eyelid with visible contour of the weight	14	No	GUE group
7	79	F	L	ANS	Gold [1.8]	extrusion	slightly reddened lid, parchment skin and very thin orbicularis muscle	8	No	GLE group
8	21	M	L	PGT	Gold [1.2]	Lid oedema	reddened and oedematous lid with visible blood vessels	21	No	GLE group
9	56	F	L	congenital	Gold [1.8]	cosmesis	pale eyelid with visible contour of the weight	61	No	GLE group
10	58	F	L	ANS	Gold [1.2]	Lid oedema	reddened and oedematous eyelid with visible blood vessels	53	No	GLE group
11	49	F	L	ANS	Gold [1.4]	Lid oedema	reddened and oedematous lid with visible blood vessels	13	Yes	GLE group
12	62	F	L	Neurofibromatosis	Gold [1.4]	extrusion	pale cicatrical eyelid	84	Yes	GLE group

*ANS* acoustic neuroma surgery, *HZO* Herpes zoster ophthalmicus, *PGT* parotid gland tumor.

GLE group = received a gold weight fixed to the tarsus 2 mm above the upper eyelid margin.

GUE group = received a gold weight fixed at the border of the upper edge of the tarsus of the upper eyelid and the levator aponeurosis.

**Figure 3:**
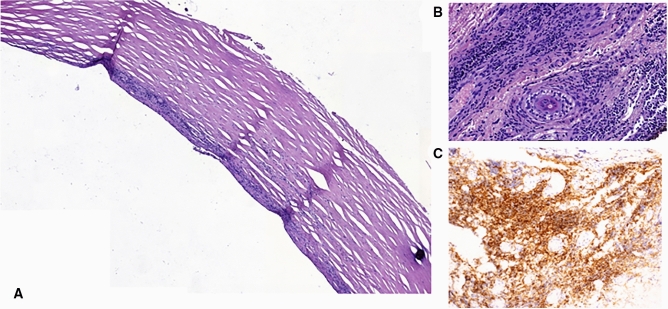
Histopathological findings after gold weight removal. (**a**) Micrograph showing a band of fibrous connective tissue with hyalinization and with visible area of lymphocytic infiltration on the surface (hematoxylin–eosin, × 20). (**b**) Micrograph showing significant inflammatory infiltration with clearly visible lymphocytes, part of the Meibomam gland and hyalinization (hematoxylin–eosin, × 20). (**c**) CD8 + cells with the CD4/CD8 ratio 1:2(DAKO, Immunoperoxidase for Dako Autostainer, × 200).

The intensity of the inflammatory infiltration differed between groups depending on the site of implantation (p < 0.001). A moderate lymphocytic reaction with the formation of the capsule consisted of hyalinized fibrous tissue around the implant was observed in 3 patients (30% of the the GLE group). Of these patients, two also experienced dilation of the surrounding blood vessels and swelling of the eyelid stroma. (Fig. 3a) In 2 other cases a small vascular proliferation was found with edema and slight fibrosis, with only single scattered lymphocytes in the stroma (the GUE group). Significant lymphocytic reaction was noted in 7 cases (70% of the GLE group). This reaction was manifested by composition of inflammatory infiltration T and B lymphocytes (CD3 + , CD20 +) with the ratio of CD4/CD8 lymphocytes = 1:2 (Fig. 3c), the presence of macrophages (CD68 + in 3/7), giant multinuclear cells around a foreign body (1/7) and the presence of remnants of other structures (5/7) such as surgical sutures (1/7), glands (2/7), epithelium, epidermis—possibly eyelid epithelium or epidermis invagination (2/7) (Fig. 3b). No other inflammatory cells were identified. Histopathology results are presented in Table 2 and Fig. 3a–c.

**Table 2:** Histopathology.

Patient	Infammatory cells	Masson trichrome	Accessory materials
1	Lymphocytes (+++), giant cell granuloma	Fibrosis	Suture, giant cells
2	Lymphocytes, macrophages	Fibrosis	Muscle
3	Lymphocytes, macrophages	Hyalinization	**–**
4	Nests of lymphocytic infiltrate	Angiogenesis	**–**
5	No inflammatory cells	Angiogenesis	Oedema
6	Few inflammatory cells, hyalinization	**–**	Pseudocyst
7	Lymphocytes in clusters, hyalinization	Angiogenesis	Hematoma
8	Lymphocytes with follicles	Focal hyalinization	**–**
9	Sparse lymphocytes, hyalinization	**–**	**–**
10	Lymphocytes (+++), fibrosis	**–**	Conjunctival epithelium
11	Lymphocytes in clusters, fibrosis	Macrophages	Glandular tubes
12	Subepidermal lymphocytic infiltrate	Hyalinization and fibrosis	Skin adnexa

### Discussion and literature review

Our study confirms that the use of gold weights induces the formation of a fibrotic capsule with a limited chronic inflammatory infiltration, which has also been reported by other authors^13,17,27,28,32,41,42^. This collagenous capsule strongly adhered to the anterior surface of the tarsal plate and by ingrowth into the fixation holes stabilized the weight in place, hence there were no muscle fibers between the implant and the tarsal plate. Serious complications which required weight removal were noted in 100% of patients in the GLE group and 20% in the GUE group during the study period (Table 1). The intensity of the inflammatory infiltration and the complication rate seem to be related to the implantation site in the upper eyelid. In all patients with extrusions and reddened swollen eyelids, the implant was sutured relatively low, about 2 mm above the eyelash line where the majority of blood vesssels and apocrine ciliary glands of Moll are located^23,44^, which may influence the intensified tissue response to the presense of the implant^41^. In a study conducted by Bair et al. an explanted weight caused no allergic reaction after placement in the inner forearm of one patient who experienced clinical signs of eyelid inflammation^28^. This may support the hypothesis that the site of implantation in the upper eyelid, rather than presumed alergic reaction, results in a clinical outcome that necessitates implant removal. The patient described by Iordanous and Evans, after initial ineffective treatment with oral antibiotics, was started on oral prednisone, which brought short-term improvement with a rapid recurrence of the symptoms after discontinuation of the steroids^30^. Topical steroids (0.5 cc of triamcinolone, 40 mg/cc) injected into the tissue of the upper eyelid resulted in complete resolution of symptoms in one of Bair’s patients who developed a sterile inflammatory reaction 1 week after gold weight implantation^28^. The same authors report antibiotic-corticosteroid ointment applied 3 weeks after surgery in the second of 3 cases of non-infectious inflammatory response to eyelid implants as totally ineffective. We also injected topical steroids into the oedematous tissue of the eyelids in patients: 8, 10, 11 at a 1-month-follow-up, but there was only partial and not long-lasting clinical improvement observed (Table 1). This may suggest that the steroid concentration might be too low to inhibit an advanced immune reaction in the upper eyelid and perhaps might have been effective at the beginning of the process. Based on the experiences of the authors mentioned above and Anderson’s study^40^ we introduced treatment with antibiotic and steroid ointment for 2 weeks as prophylaxis against infection and possible immune reaction to the implant at the initial stage.

To our knowledge there are only 8 papers discussing histopathological findings after gold weight removal^13,17,23,27,28,32,41,42^. In each study conducted by Seiff et al.^13^, Townsend DJ ^17^, Doyle et al.^27^ and Kilduff et al.^32^ there is only a single specimen evaluated histopathologically. Bair et al. report histopathological findings of 3 specimens^28^. Schrom et al. describe the results of an examination of 10 samples harvested after loading the upper eyelid with gold weights and of 8 samples after platinum chain insertion, but there are no detailed clinical data described. He suggests that it is impossible to foresee the exact tissue reaction to the implant because of the co-existence of many modifying factors which vary among individuals^42^. We acknowledge the fact that our study has limitations, including restricted immunohistochemical examination and lack of patch testing. The collected material does not allow for conducting sophisticated statistical analyses due to small sample size. In the PUE group the patient with reddened eyelid refused to have the implant removed from the eyelid tissues, thus no platinum chain was examined histopathologically. Nevertheless, from the cases we have observed, apart from obvious individual variability, a certain pattern of undesirable reactions to gold implants emerges. These reactions can be divided into two distinct types depending on the implantation site:

1. Slight to moderate lymphocytic reaction with the formation of the capsule consisted of hyalinized fibrous tissue around the implant—characteristic and observed in other parts of the body when implanting biologically inert materials. We observed this type of moderate lyphocytic reaction in 3 cases, 2 of which were accompanied by dilation of the surrounding blood vessels and swelling of the eyelid stroma (30% explanted weights in the GLE group).
  In 2 other cases only small vascular proliferation was found with edema and slight fibrosis, with only single scattered lymphocytes in the stroma (100% explanted weights in the GUE group) (Fig 3A).
2. Significant lymphocytic reaction (7 cases, 70% explanted weights in the GLE group, 70% of the GLE group), composed of inflammatory infiltration T and B lymphocytes (CD3 + , CD20 +) with the ratio of CD4/CD8 lymphocytes = 1:2 (Fig. 3C), the presence of macrophages (CD68 + in 3/7), giant multinuclear cells around a foreign body (1/7) and the presence of remnants of other structures (5/7) such as surgical sutures (1/7), glands (2/7), epithelium, epidermis—possibly eyelid epithelium or epidermis invagination (2/7) (Fig. 3B).

This study confirms that weight placement site in the upper eyelid may be critical for procedure success. We strongly believe that by providing detailed information about past medical histories, conducted examinations, weights used, surgical technique, postoperative care plan and histopathological findings in every case, our study may constitute a step further in the process of comprehending the possible impact of gold weights on the upper eyelid microenviroment in patients with unresolved FP.

## Conclusion

Placement of gold weight in the upper eyelid induces a limited chronic inflammatory response of varied intensity and local fibrosis which helps to stabilize the implant in place and may reduce its biological activity. Gold weights produce only a slight reaction while remaining biologically inert. Fibrosis and single scattered lymphocytes seem to indicate a reaction to the mechanical irritation of the eyelid tissues, which may be accompanied by the widening of small blood vessels and swelling of the stroma. The severe reactions we observed after placement of weights close to the lid margin are instead caused either by the type of auxiliary materials used (sutures) or by dragging other tissues into the area around the implant (the observed inflammatory reactions are largely consistent with those observed in chronic inflammation of the eyelid glands [composed of inflammatory infiltrate, macrophages, giant cells]). It seems, therefore, that the decisive factor in such reactions to gold implants is mechanical damage to the eyelid structures, and not the material of the weight, itself. The site of weight placement in the upper eyelid may be critical for procedure success. Upper implantation of weights seems to cause less damage to the eyelid tissues. Placement of the gold weights about 2 millimeters above the eyelash line with absorbable sutures should be avoided due to the high risk of complications.

## Data Availability

The datasets used and/or analysed during the current study are available from the corresponding author upon request.

## References

[1] Slattery WH, Azizzadeh B (2014). The facial nerve.

[2] Nowak-Gospodarowicz I, Rękas M (2021). predicting factors influencing visual function of the eye in patients with unresolved facial nerve palsy after upper eyelid gold weight loading. J. Clin. Med..

[3] Nowak-Gospodarowicz I, Rozycki R, Rekas M (2020). Quality of life in patients with unresolved facial nerve palsy and exposure keratopathy treated by upper eyelid goldweight loading. Clin. Ophthalmol..

[4] Illig KM (1958). Eine neue operationsmethode gegen laophthalmus. Klin Monatsbl Augenheilkd.

[5] Smellie GD (1966). Restoration of the blinking reflex in facial palsy by a simple lid-load operation. Br PlastSurg..

[6] Sheehan JE (1950). Progress in correction of facial palsy with tantalum wire and mesh. Surgery..

[7] Morel-Fatio D, Lalardrie JP (1964). Palliative surgical treatment of facial paralysis: the palpebral spring. Plast Reconstr Surg..

[8] Lessa S, Carreirão S (1978). Use of an encircling silicone rubber string for the correction of lagophthalmos. Plast Reconstr Surg..

[9] May M (1987). Gold weight and wire spring implants as an alternative to tarsorrhaphy. Arch. Otolaryngol..

[10] May M, Drucker C (1993). Temporalis muscle for facial reanimation: A 13 year experience with 224 procedures. Arch. Otolaryngol. Head Neck Surg..

[11] Berghaus A, Neumann K, Schrom T (2003). The platinum chain: A new upper-lid implant for facial palsy. Arch. Facial Plast. Surg..

[12] Jobe RP (1974). A technique for lid loading in the management of the lagophthalmos of facial palsy. Plast. Reconstr. Surg..

[13] Seiff SR, Sullivan JH, Freeman LN, Ahn J (1989). Pretarsal fixation of gold weights in facial nerve palsy. Ophthal. Plast. Reconstr. Surg..

[14] Cies WA (1993). Modified gold weights for reanimation of the upper lid in facial nerve paralysis. Ophthal. Plast. Reconstr. Surg..

[15] Geoffrey J, Gladstone GJ, Nesi FA (1996). Management of paralytic lagophthalmos with a modified gold-weight implantation technique. Ophthal. Plast. Reconstr. Surg..

[16] Caesar RH, Friebel J, McNab AA (2004). Upper lid loading with gold weights in paralytic lagophthalmos: A modified technique to maximize the long-term functional and cosmetic success. Orbit.

[17] Townsend DJ (1992). Eyelid reanimation for the treatment of paralytic lagophthalmos: Historical perspectives and current applications of the gold weight implant. Ophthal. Plast. Reconstr. Surg..

[18] Rahman I, Sadiq SA (2007). Ophthalmic management of facial nerve palsy: A review. Surv. Ophthalmol..

[19] Schrom T (2007). Lidloading in facial palsy. Laryngorhinootologie..

[20] Pausch N, Sterker I, Hemprich A, Frerich B (2006). Restoration of lid function in peripheral facial palsy by implanting gold weights. Mund Kiefer Gesichtschir..

[21] Sönmez A, Oztürk N, Durmus N, Bayramiçli M, Numanoglu A (2008). Patients’perspectives on the ocular symptoms of facial paralysis after gold weight implantation. J. Past. Reconstr. Aesthet. Surg..

[22] Sadiq SA, Downes RN (1998). A clinical algorithm for the management of facial nerve palsy from an oculoplastic perspective. Eye.

[23] Amer TA, El-Minawi HM, El-Shazly MI. Low-level versus high-level placement of gold plates in the upper eyelid in patients with facial palsy. *Clin. Ophthalmol.* 2011; 5 891– 895 10.2147/OPTH.S21491 PMC3133007 21760718

[24] Aggarwal E, Naik MN, Honvar SG (2007). Effectiveness of the gold weight trial procedure in predicting the ideal weight for lid loading in facial palsy a prospective study. Am J Ophtalmol..

[25] Pickford MA, Scamp T, Harrison DH (1992). Morbidity after gold weight insertion into the upper eyelid in facial palsy. Br. J. Plast. Surg..

[26] Schrom T, Wernicke K, Helen A, Knipping S (2007). Results after lidloading with rigid gold weights-a meta-analysis. Laryngorhinootologie..

[27] Doyle E, Mavrikakis I, Lee EJ, Emerson R, Rainey AJ, Brittain GP (2005). TypeIV hypersensivity reactions to upper lid gold weight implants-Is patch testing necessary?. Orbit.

[28] Bair RL, Harris GJ, Lyon DB, Komorowski RA (1995). Noninfectious inflammatory response to gold weight eyelid implants. Ophthal. Plast. Reconstr. Surg..

[29] Dinces EA, Mauriello JA, Kwartler JA, Franklin M (1997). Complications of gold weight eyelid implants for treatment of fifth and seventh nerve paralysis. Laryngoscope..

[30] Iordanous Y, Evans B (2012). Noninfectious inflammatory reaction to a gold weight eyelid implant: A case report and literature review. Can. J. Plast. Surg..

[31] Björkner B, Bruze M, Möller H, Salemark L (2008). Allergic contact dermatitis as a complication of lid loading with gold implants. Dermatitis.

[32] Kilduff CLS, Casswell EJ, Imonikhe R, Marjanovic B (2018). Type IV hypersensitivity to gold weight upper-eyelid implant: Case report and review of the literature. Ocul. Immunol. Inflamm..

[33] Ritz M, Southwick GJ, Greensmith A, Gory I (2006). Gold sensitivity after gold weight eyelid insertion for facial palsy. Aesth Plast Surg..

[34] Fowler JF (1987). Selection of patch test materials for gold allergy. Contact Dermatitis.

[35] Bruze M, Andersen KE (1999). Gold-a controversial sensitizer. Contact Dermatitis.

[36] Björkner B, Bruze M, Möller H (1994). High frequency of contact allergy to gold sodium thiosulfate: An indication of gold allergy?. Contact Dermatitis.

[37] Anhilide I, Björkner B, Bruze M, Möller H (2000). Exposure to metallic gold in patients with contact allergy to gold sodium thiosulfate. Contact Dermatitis.

[38] Martin SF (2015). New concepts in cutaneous allergy. Contact Dermatitis.

[39] Martin SF (2014). Adaptation in the innate immune system and heterologous innate immunity. Cell Mol. Life Sci..

[40] Anderson JM, Rodriguez A, Chang DT (2008). Foreign body reaction to biomaterials. Semin. Immunol..

[41] Nowak-Gospodarowicz I, Rozycki R, Koktysz R, Rekas M (2017). Complications associated with the surgical techniques of upper eyelid loading: A clinicopathologic study of 7 explanted gold weight lid loads. Int. J. Ophthalmic. Pathol..

[42] Schrom T, Taege C, Wolf G, Reinhardt A, Scherer H (2006). Histopathologie nach implantation von Lidgewichten. HNO.

[43] Zide BM, Jelks GW (1985). Surgical anatomy of the orbit.

[44] Forrester JV, Dick AD, McMenamin PG, Roberts F. Anatomy of the eye and orbit. In: Forrester JV. *The eye: Basic sciences in practice*, London: WB Saunders;3e, 2008:ch.1, p.86–88.

[45] Kelly SA, Sharpe DT (1992). Gold eyelid weights in patients with facial palsy: a patients review. Plast. Reconstr. Surg..

[46] Comprehensive Jewelry Precious Metals Overview. International Gem Society (IGS), Retrieved 01–16–2015.

[47] World Gold Council (2003) About gold jewellery, Online article accessed 8 October 2014.

[48] Deziel-Evans LM, Hussey WC (1989). Possible sulfite sensitivity with gentamicin infusion. Drug Intell. Clin. Pharm..

